# A home-based pulmonary rehabilitation mHealth system to enhance the exercise capacity of patients with COPD: development and evaluation

**DOI:** 10.1186/s12911-021-01694-5

**Published:** 2021-11-22

**Authors:** Ning Deng, Leiyi Sheng, Wangshu Jiang, Yongfa Hao, Shuoshuo Wei, Bei Wang, Huilong Duan, Juan Chen

**Affiliations:** 1 College of Biomedical Engineering and Instrument Science, Ministry of Education Key Laboratory of Biomedical Engineering, Zhejiang University, Hangzhou, China; 2 Alibaba-Zhejiang University Joint Research Center of Future Digital Healthcare, Hangzhou, China; 3 Department of Pulmonary and Critical Care Medicine, General Hospital of Ningxia Medical University, Yinchuan, 750004 Ningxia China

**Keywords:** COPD, Pulmonary rehabilitation, mHealth, Behavior change wheel

## Abstract

**Background:**

Patients with chronic obstructive pulmonary disease (COPD) experience deficits in exercise capacity and physical activity as their disease progresses. Pulmonary rehabilitation (PR) can enhance exercise capacity of patients and it is crucial for patients to maintain a lifestyle which is long-term physically active. This study aimed to develop a home-based rehabilitation mHealth system incorporating behavior change techniques (BCTs) for COPD patients, and evaluate its technology acceptance and feasibility.

**Methods:**

Guided by the medical research council (MRC) framework the process of this study was divided into four steps. In the first step, the prescription was constructed. The second step was to formulate specific intervention functions based on the behavior change wheel theory. Subsequently, in the third step we conducted iterative system development. And in the last step two pilot studies were performed, the first was for the improvement of system functions and the second was to explore potential clinical benefits and validate the acceptance and usability of the system.

**Results:**

A total of 17 participants were enrolled, among them 12 COPD participants completed the 12-week study. For the clinical outcomes, Six-Minute Walk Test (6MWT) showed significant difference (*P* = .023) over time with an improvement exceeded the minimal clinically important difference (MCID). Change in respiratory symptom (CAT score) was statistically different (*P* = .031) with a greater decrease of − 3. The mMRC levels reduced overall and showed significant difference. The overall compliance of this study reached 82.20% (± 1.68%). The results of questionnaire and interviews indicated good technology acceptance and functional usability. The participants were satisfied with the mHealth-based intervention.

**Conclusions:**

This study developed a home-based PR mHealth system for COPD patients. We showed that the home-based PR mHealth system incorporating BCTs is a feasible and acceptable intervention for COPD patients, and COPD patients can benefit from the intervention delivered by the system. The proposed system played an important auxiliary role in offering exercise prescription according to the characteristics of patients. It provided means and tools for further individuation of exercise prescription in the future.

**Supplementary Information:**

The online version contains supplementary material available at 10.1186/s12911-021-01694-5.

## Background

Chronic obstructive pulmonary disease (COPD) is characterized by airflow limitation and increasing respiratory symptoms and a progressive disabling respiratory and systemic condition [1]. According to the most recent Chinese national survey of COPD during 2012–2015, the overall prevalence of COPD was 14% among people aged 40 years or older [2]. Even with smoking cessation and pharmacological treatment, patients with COPD experience deficits in exercise capacity and physical activity as their disease progresses [3, 4]. It is therefore crucial for patients to maintain a lifestyle which is long-term physically active to increase their exercise capacity, reduce dyspnea and improve their health-related quality of life (HRQL) [1, 5]. A recent meta-analysis of 10 randomized controlled trials demonstrated that PR, when compared with usual care, is associated with lower overall rates of hospitalizations from acute exacerbations of COPD [6]. Pulmonary rehabilitation (PR) is a comprehensive intervention including physical exercise training, patient education, and nutritional/psychosocial support. global initiative for chronic obstructive lung disease (GOLD) guidelines emphasized that COPD rehabilitation had been recognized to be an evidence-based treatment recommended for COPD [7, 8]. Despite the well-documented benefits of PR, long-term access and utilization of PR by COPD patients remains low due to insufficient funding, resources, and other patient-related barriers limiting long-term access to PR [9]. In China, despite clinical guideline recommendations, poor perception, low referral and limited uptake rates were common among COPD patients [10] in addition to poor capability among primary health care providers (HCPs), and inconvenience regard to time or transportation [10, 11]. Polkey et al. conducted a clinical trial that compared Tai Chi with conventional PR in COPD patients, the results showed that Tai Chi is equivalent to PR for improving SGRQ in COPD and demonstrated that Tai Chi is an appropriate substitute for PR in COPD in China [12]. Ambrosino and colleagues have suggested that although Tai Chi may have the potential of providing a low-cost initial therapy among patients with COPD in China, Tai Chi recreational exercise is not rehabilitation [13]. There is thus an urgent need for promotion of PR, and for further development of more convenient and accessible strategies of PR to extend the initial benefits of PR in COPD patients.

At present, mHealth technology provides a convenient and low-cost approach to support home-based disease management, mHealth technology allowing all intervention components to be delivered at home with proven clinical outcomes, especially in remote and rural areas [14]. Specifically, smartphones enable HCPs to monitor patients anytime and anywhere, and the smartphone applications (“apps”) are considered as a potentially promising approach for behavior change [15]. Rassouli et al. have examined the effect of a smartphone app (Kaia COPD) which digitized PR in COPD. They found that digitalizing PR is feasible and accepted and that a short-term improvement of HRQoL was achieved [16]. Kwon et al. developed a comprehensive rehabilitation platform and found significant improvement in HRQoL [17]. However, Kwon et al. found no significant improvement in exercise capacity (with 6-min walk test, 6MWT). Although previous studies have examined the effect of home-based PR using apps in COPD patients, they resulted in limited conclusions.

Even some studies have shown that Home-based PR with interventions for daily life might be helpful to enhance access and uptake [18], achieving a more prolonged long-term effect and helping patients adopt a more active lifestyle still remains a significant challenge [19, 20]. There is some evidence that interventions incorporating behavior change technologies (BCTs) could lead to better engagement in PR and maintenance of behavior change [21]. Relatively few studies have mapped BCTs into interventions to enhance the effect of a comprehensive program for patients with COPD. The study by Burkow and colleagues evaluated the impact of BCT-based tablet intervention on physical activity in COPD patients [22]. In another study, Bentley et al. developed a mHealth intervention (SMART-COPD) delivered via a smartphone app and an activity tracker, to help people with COPD maintain physical activity after undertaking PR [23]. Both studies focused on physical activity improvement and the acceptance of intervention while no other clinical outcomes were available.

Thus far, researchers have used mHealth technology and behavior change theory to perform home-based PR. Nevertheless, studies to explore the method of maintaining a long-time effectiveness of home-based PR have remained comparatively lacking. Limited information has been available on the effect of a mHealth-based, theory-guided PR program for COPD patients at home. Home-based PR program using apps may have different effect and this innovative intervention bears investigation with COPD patients to assess its full potential.

Based on literature research two assumptions were made. The first was that delivering PR through mHealth technology is feasible and acceptable for participants of pulmonary rehabilitation, and participants can benefit from such mode PR [24]. The second was that it is advantageous to design interventions that are underpinned by behavior change theories or framework, e.g., the behavior change wheel (BCW) [25]. To further investigate the method for designing a mHealth-based, theory driven PR, this study aimed (1) To develop a home-based rehabilitation mHealth system incorporating behavior change techniques (BCTs) for COPD patients, and (2) To evaluate its technology acceptance and feasibility.

## Methods

### Study setting and ethical considerations

Two pilot studies have been conducted in this study (hereinafter referred to as “preliminary test” and “assessment test” respectively). Both of them were conducted at the General Hospital of Ningxia Medical University in collaboration with Zhejiang University, China, from 2020 to 2021. And they were approved by the Ethics Committee for the Conduct of Human Research at General Hospital of Ningxia Medical University (2020-338). All the participants signed an informed consent. All methods were performed in accordance with relevant guidelines and regulations.

### Participants

Patients with COPD were recruited from outpatient clinics according to the following inclusion criteria: (1) eligible patients were aged between 40 and 80 years; with a diagnosis of COPD (post-bronchodilation forced expiratory volume in 1 s (FEV1)/forced vital capacity (FVC) < 70%) with moderate to severe airflow limitation and under optimal medical treatment according to the global initiative for chronic obstructive lung disease (GOLD) [1] and a history of acute exacerbations of COPD 1 year prior to entering the study according to our previous study [14] (2) ability to walk > 150 m in a 6MWT; and (3) a smartphone owner. Patients who were unable to use WeChat mini program were excluded from the screening process.

### Intervention

Patients involved in the intervention used the app developed in this study to conduct PR at home for several weeks (8 weeks in preliminary test, and 12 weeks in assessment test), during which their activities and exercise data were monitored and collected. All participants received personalized exercise prescription during the intervention. The initial intensity of exercise prescription was determined by the participant’s baseline level of activity. Patients enrolled were trained by experienced HCPs about the correct use of the app. In addition, we provided a range of portable devices for each one and made sure that they can be correctly used by patients themselves.

Usual care and treatment: included optimal medical treatment and pharmacotherapy oxygen therapy if presence of respiratory failure. Furthermore, patients were tracked an acute exacerbation of COPD events according to our previous report [14].

### Assessment and data collection

An assessment of COPD patients before beginning and in the end of the exercise training program were conducted during this PR process, which including indirect peripheral oxygen saturation measured by oximetry (SpO_2_), and dyspnea monitoring using the Borg scale. During the process of PR, SPO_2_% and heart beat rate was monitored during exercise and SpO_2_ is ≤ 88% at room air breathing or heart beat rate more than 130 per minute were recommendation for cessation exercise.

In the preliminary test, patients’ 6-Minute Walking Test (6MWT) outcome was measured and participants’ and HCPs’ opinions were collected in order to better meet the needs of users.

In the assessment test, the primary clinical outcome was the change in the 6MWT. Secondary clinical outcomes included changes in COPD Assessment Test (CAT), mMRC (modified British medical Research Council), Hospital Anxiety and Depression Scale (HAD), Chronic Respiratory Questionnaire (CRQ) and Clinical COPD Questionnaire (CCQ). For these self-reported parameters, patients were asked to fill out a questionnaire both at the beginning of the study period and after completing exercise sessions through the app.

In addition to what have been mentioned above, compliance, technology acceptance, and feasibility of the system were also used as outcome indicators. Compliance was defined as the ratio of the actual frequency of self-reported exercise records to the requirement. To measure the participants’ acceptance, we performed a short survey with an 11-item questionnaire extracted from our previous study [26] which used a 5-point Likert scale. As for feasibility, a 4-item usability questionnaire was used (see Additional file 1). Semi-structured interviews with open-ended and closed questions were conducted to further explore patients' attitudes, guided by the Focused Conversation Method (also named ORID method). Examples of the questions used during the interview were listed in Additional file 2.

Data were collected at baseline, during intervention, and end of intervention. A summary was shown in Table 1.

**Table 1:** Detail of measures to be collected

Type of outcomes	Preliminary test^a^	Assessment test^b^	Collection time
*Quantitative*			
Clinical outcomes	6MWT	6MWT, CRQ, CCQ, CAT, mMRC, HAD	Baseline, end of intervention
Compliance	C_i_^c^	C_i_^c^	During intervention
Technology acceptance	None	11-Item questionnaire	End of intervention
Feasibility	None	4-Item questionnaire	End of intervention
*Qualitative*			
Subjective opinion	Interview	Interview	End of intervention

*6MWT* six-minute walk test, *CRQ* chronic respiratory questionnaire, *CCQ* clinical COPD questionnaire, *CAT* COPD assessment test, *mMRC* modified British medical research council, *HAD* Hospital Anxiety and Depression Scale

^a^Preliminary test: This column listed the measures used in the first polit study called “Preliminary test”

^b^Assessment test: This column listed the measures used in the second polit study called “Assessment test”

^c^C_i_: min($\frac{actual\;duration\;of\; exercise\;in\;one\;day}{{required\;duration\;of\;exercise\;in\;one\;day}},1$), the formula used for calculating compliance referred from [27]

### Development of interventions

The design process of the intervention was composed of two phases, combining key elements of the Medical Research Council (MRC) framework, which was often used for the development and evaluation of complex clinical interventions in health care [28]. This framework recommends the use of an iterative, phased approach that harnesses qualitative as well as quantitative methods to improve study design.

**Fig. 1:**
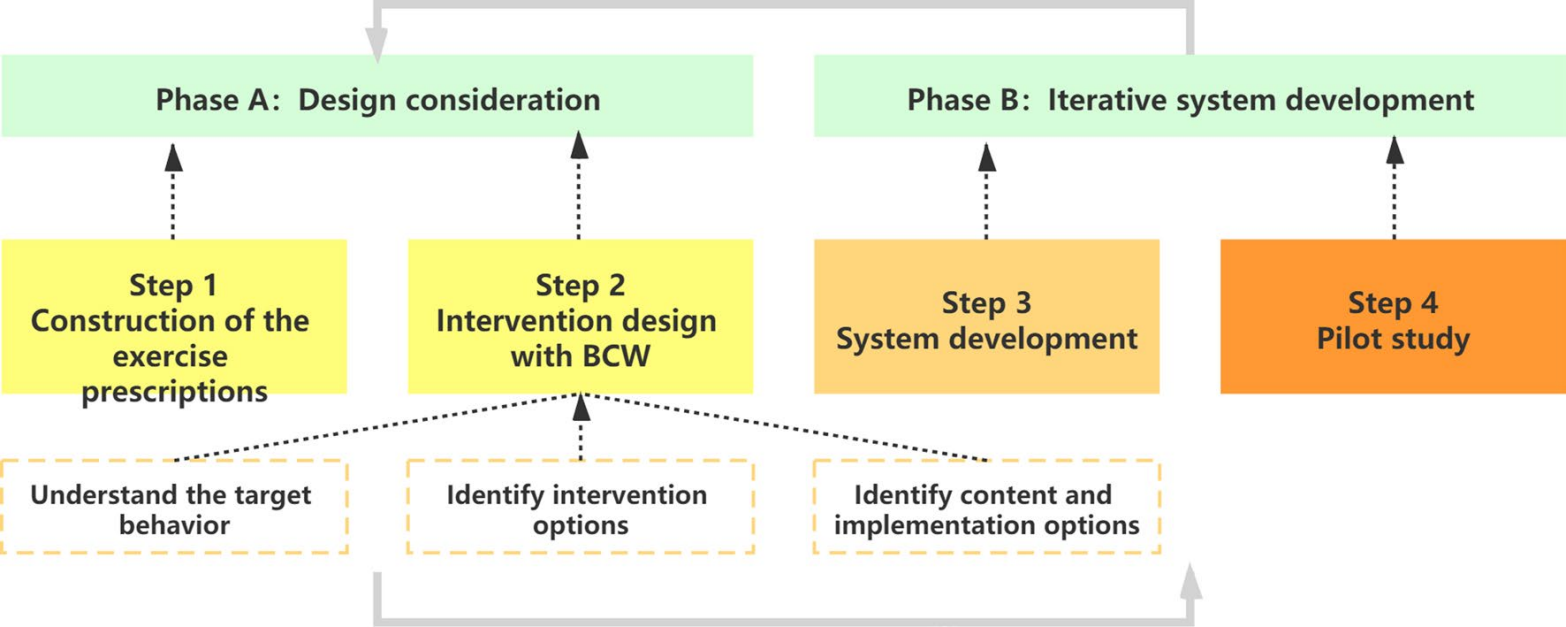
Schematic describing the design process of home-based PR incorporating BCW guided by the MRC framework. *BCW* behavior change wheel, *MRC* medical research council

The methods of each step are chronologically described in the following subsections in detail and Fig. 1 illustrates the whole study process. The results of each step are detailed in the “Results” section.

#### Phase A: DESIGN consideration

##### Step 1: Construction of exercise prescription

Considering the following points, the first step aimed to identify the contents and structure of exercise prescription suitable for home-based PR given the following considerations. Firstly, clinical guidelines for center-based PR cannot be applied to home-based PR directly as patients at home lack training equipment and supervision of professional HCPs. It is necessary to provide patients at home with exercise prescription that is as detailed as possible, easy-to-understand and independently executable without professional equipment. Secondly, the structure of the exercise prescription needs to be adapted to mHealth technology to facilitate the efficiency of the interaction and storage of information. Thirdly, COPD is a complex and individual heterogeneous disease with which patients need individualized and tailored treatment [29].

Guiding principles for the exercise prescription were formulated at an early stage to provide a framework for the construction of exercise prescription. It was clarified that exercise prescriptions for home-based PR should: (1) be evidence-based and meanwhile be straightforward and visually, easy for the elderly to understand; (2) be personalized. The comprehensive intervention composed of patient tailored therapies should be formulated based on a thorough patient assessment [30]; (3) take full account of environmental constraints such as safety and lack of equipment.

##### Step 2: Intervention design with BCW

The core of BCW is COM-B (capability, opportunity, and motivation–behavior) model. It points out that for an individual the occurrence of certain behavior such as conducting resistance training at home depends on three conditions: capability, opportunity, and motivation [31]. For example, a patient may not take upper limb training at home because he or she is neither aware of the benefits nor aware of how to carry out. Based on the definition of this framework, this patient is lacking the motivation and capability to perform the behavior. In this case, behavioral intervention can be taken against the factors that hinder the behavior to occur. The patient's motivation can be improved through health education by doctors, and the patient ' s ability to take exercise can be given by displaying animation containing training action guidance on the mobile phone. Around the COM-B model, there are nine intervention functions to choose. In addition, seven types of strategies are provided as the method to implement these intervention functions.

The process of intervention development has been summarized into three stages [32]. In the first phase, target behavior needed to be fully understood and defined using terminology. Then analysis was performed to identify what needs to change in patients’ capacity, opportunity, and motivation to improve target behaviors. Finally based on behavior analysis of these behaviors, potential intervention functions and BCTs were selected for the intervention. Moreover, the design requirements for intervention components were formulated by mapping out the potential working BCTs.

#### Phase B: Iterative system development

##### Step 3: System development

To implement the aforementioned design, we had proposed a system that contains three parts: an app for patients, a workstation for healthcare providers, and cloud server. The app for patients would be implemented using WeChat mini programs, which are easy to access and share, suitable for both iOS and Android.

Phase B consists of two cycles. In the first cycle, we built initial mockups as the first version of the system and tested them with patients with COPD. Private interview was used to measure the most valuable features of the system for users, focusing on several key aspects of availability and feasibility. For problems found in the first cycle, we made corresponding improvements to the system in the second cycle.

##### Step 4: Pilot study

Two pilot studies were performed, the first was for the improvement of system functions and the second was to explore potential clinical benefits and validate the acceptance and usability of the system. Information on participants, interventions, and study setting were described in the corresponding section of the “Method” part above.

### Statistical analysis

Data preprocessing were performed using Python 3.7 and data were analyzed with SPSS version 24.0 (IBM Corp). For the primary clinical outcome 6MWT, a change between 25 and 33 m is considered a minimal clinically important difference (MCID) in patients with COPD [33]. Differences between the full intervention period in primary and secondary outcomes were analyzed using T-test or Wilcoxon signed-rank test. *P* values of less than 0.05 were considered statistically significant. For the qualitative analysis, interviews were audiotaped, and transcribed verbatim by YfH. LyS conducted the analysis process during which patients’ description was summarized to patients’ perceptions [34], and quotes from the interviews had been translated into English.

## Results

### System description

#### Step 1: Construction of exercise prescription

Based on the characteristics of prescription proposed in the method section (phase A, step 1), three key elements of exercise prescription were defined: structure, content, and principle.

**Fig. 2:**
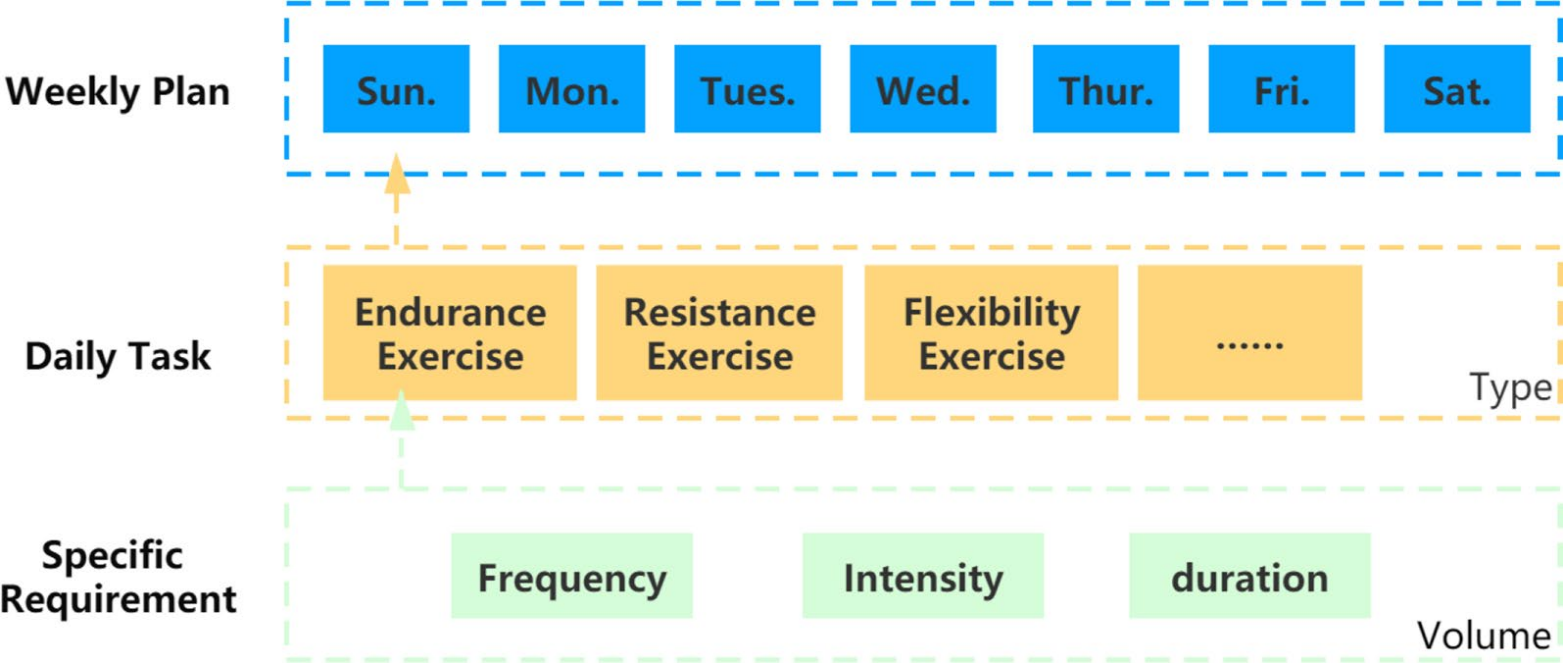
Structure of exercise prescription

A three-level structure (Weekly plan—Daily task—Specific requirement) was adopted as Fig. 2 shows. Overall, patients' exercise prescription was formulated weekly. If no adjustments are required, the prescription will be repeated weekly.

Indeed, the structure of prescription followed the F.I.T.T principle (frequency, intensity, time, and type of exercise) [35]. But we re-organized the construction form into two dimensions: type, which is equivalent to the type of FITT, and volume that contains frequency, intensity, and time (rename as duration) [35].

For the content, we had consulted three current evidence-based guidelines related to pulmonary rehabilitation, including the American Thoracic Society/European Respiratory Society (ATS/ERS) [7], the American College of Sports Medicine (ACSM) [35], the Australia Lung Foundation [36], etc. [37, 38]. Combined them with opinions from the practice-based experience of the HCPs from our team, exercises that were accessible in the home environment were selected. These exercises were summarized suitable for the structure proposed above and were listed in Table 2.

**Table 2:** Content of exercise prescription designed in this study

Type		Volume		
Main type	Subtype	Frequency	Duration	Intensity
Aerobic/endurance training	Walk	3–7 days per week	10–60 min per day;	measured by the walk rate
Resistance training	Upper limbs^a^	2–5 days per week	2–12 repetitions per set; 1–3 sets per day; held for 30 to 60 s per repetition; 2–4 repetitions per set; 1–2 sets per day	measured by subjective feelings of patients indicated by Borg scale value between 4–6
	Lower limbs^b^			
Flexibility training	Shoulder stretch, side stretch, thoracic stretch	2–3 days per week		
Respiratory muscle training	calm and natural breathing, abdominal breathing	3–7 days per week	1–9 min per set; 2 sets per day	

^a^Upper limbs resistance training include: bicep curl, shoulder press, side lateral raise, and wall push up referred from Ref. [36]

^b^Lower limbs resistance training include: sit to stand, step ups, heel raises, leg extension, and seated row referred from Ref. [36]

The principles to be followed in the formulation of prescriptions contained four points, which emphasized prescriptions for patients should (1) be assessment based. We used 6MWT for assessing functional exercise capacity, for severe COPD patient, a typically walking test for 10 min was used for graded exercise testing. (2) Contain various but targeted types. For example, for patients with exercise intolerance, endurance training were given; for patients with insufficient upper limb muscle strength, upper limb resistance training strength were included. (3) Keep progressive overload volume [39]. Taking aerobic exercise training as an example, the frequency can be set to 7 sessions per week, the duration of exercise is 20 min per once, 80% of the intensity of exercise of peak work rate were given at initial and were adjusted every 2 weeks by HCPs manually. (4) Ensure safety. SPO_2_% and heart beat rate was monitored during exercise and SpO_2_ is ≤ 88% at room air breathing or heart beat rate more than 130 per minute were recommendation for cessation exercise.

#### Step 2: Intervention design with BCW

During the step of intervention design, this study decided to focus on these two target behaviors: (1) exercise behavior of COPD patients at home to improve their exercise capability and physical activity (the primary target behavior) (2) supervision and management behavior of healthcare providers, including prescriptions adjustment and follow up (the secondary target behavior).

Each target behavior was analyzed to determine what needs to be changed and how to promote the occurrence of behavior change using the COM-B model. Table 3 outlines the results of analysis and different potential intervention functions associated with the corresponding COM-B components identified to facilitate the target behavior. Specifically, for exercise behavior of COPD patients at home, all COM-B components needed to change; for supervision and management behavior of healthcare providers, opportunity and motivation needed to change. Five out of nine intervention functions were identified: *Training, Education, Enablement, Persuasion,* and *Incentivization.* Concerning policy categories, only two out of the seven categories highlighted in the BCW guide were identified. These included *Communication/marketing* and *Service provision.*

**Table 3:** Behavior analysis and diagnosis

COM-B component	Target behavior	Conditions required for the target behavior to occur	Need to change or not	Potential candidate intervention functions
Physical capability	PA^a^	Patients should have the physical skills and fitness for home-based PR	Yes	Training enablement
	HP^b^	HCPs have sufficient professional ability	No	/
Psychological capability	PA	Patients should know the correct technique to perform exercises and skills to be physically active	Yes	Education
	HP	HCPs have acquired relevant knowledge	No	/
Physical opportunity	PA	Create the opportunity to be perform exercise	Yes	Enablement
	HP	Create the opportunity to access patients’ exercise data	Yes	Enablement
Social opportunity	PA	See members in close social networks valuing physical activity	Yes	Enablement
	HP	Unrelated	No	/
Reflective motivation	PA	Patients should hold beliefs that being physically active benefits their health	Yes	Education persuasion
	HP	Unrelated	No	/
Automatic motivation	PA	Create established routines and habits for physical activity	Yes	Persuasion incentivization
	HP	Create established routines and habit for supervision and management of exercise related data	Yes	Persuasion incentivization

^a^PA: Exercise behavior of COPD patients at home

^b^HP: Supervision and management behavior of healthcare providers

After selecting the intervention functions and policy categories that might contribute to the implementation of the intervention, two members of our research team jointly identified the BCTs to develop the final model of our system, which might be best to serve the previously identified BCW components. As a result, 18 out of the 93 BCTs in the BCT taxonomy version 1 were identified, resulting in 13 design requirements of the intervention (Table 4). The BCT taxonomy version 1 with the 18 identified BCTs marked with * can be found in Additional file 3.

**Table 4:** BCTs identified and design requirements for mHealth intervention

Potential candidate BCTs	Intervention components	Design requirements for the system
1.1 Goal setting (behavior)	Personalized exercise prescription module (for patients)	Set goals and deliver exercise prescriptions for patients using the app. (1.1, 1.4)
1.4 Action planning		
4.1 Instruction on how to perform a behavior		Provide step-by-step instructions on how to perform an exercise for patients, such as videos, audios and images. (4.1, 6.1, 8.1)
6.1 Demonstration of the behavior		Tell patients what to do directly. (7.1)
7.1 Prompts/cues		Exercise prescription should be graded and stepwise in intensity. (8.7)
8.1 Behavioral practice/rehearsal		Provide portable devices such as activity tracker. (12.5)
8.7 Graded tasks		
12.5 Adding objects to the environment		
1.2 Problem solving	Control of management process	Self-monitor and record exercise-related data using the app. (2.3)
1.5 Review behavior goal(s)		Support from family or HCPs. (3.1)
1.6 Discrepancy between current behavior and goal		Review and compare exercise-related records uploaded with the prescribed plan; Feedback the comparison results to patients; Modify the personalized exercise prescription according to the results. (1.5, 1.6, 2.2)
2.2 Feedback on behavior		
2.3 self-monitoring of behavior		
3.1 Social Support (unspecified)		Solve problems in the home-based PR process. (1.2)
2.7 Feedback on outcome(s) of behavior	Education and feedback (for HCPs)	Follow-up and feedback the health-related changes during this time. (2.7)
5.1 Information about health consequences		Provide counseling by HCPs or health education to inform patients of the benefits of PR. (5.1)
10.4 Social reward		Reward patients with high compliance. (10.4)
12.1 Restructuring the physical environment		Provide technical support for HCPs with mHealth. (12.1)

#### Step 3: System development

**Fig. 3:**
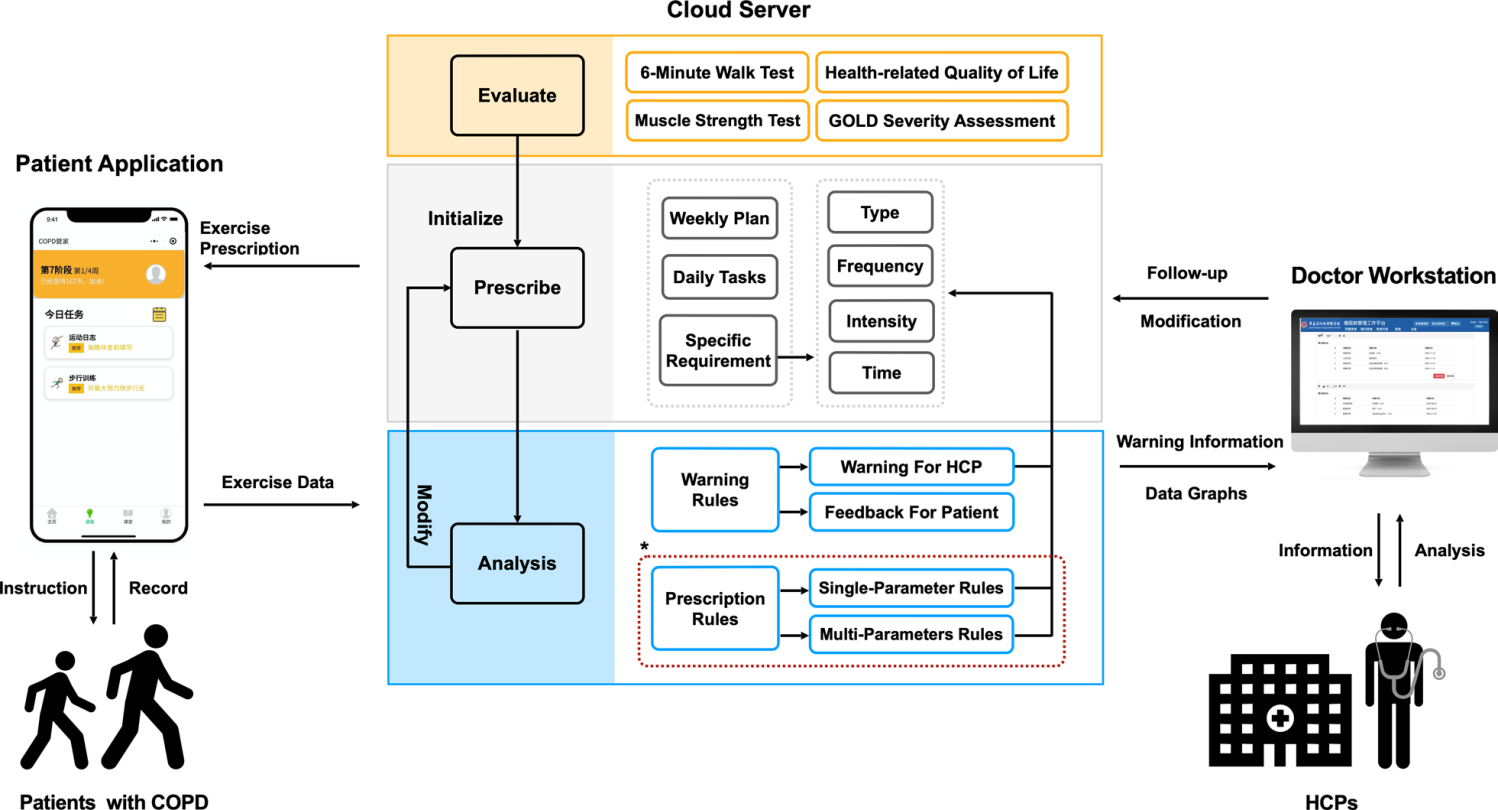
The conceptual model of the proposed system and the intervention process

Based on design requirements, the conceptual model for intervention and content of the system were developed. Figure 3 shows an overview of the conceptual model. Patients were first evaluated to identify their initial physical capacity and disease severity. Then personalized exercise prescriptions were provided and patients would perform these exercises. Exercise-related data will be received by the rule engine, which provides decision support for HCPs. The rule engine will also provide patients with different types of feedback to motivate and authorize them to achieve their goals. Exercise data and early warning information will also be sent to HCPs who will adjust their prescriptions according to patient conditions.

Iterative system development resulted in a functional mobile app for patients to (1) receive daily tasks of exercise prescription; (2) undertaken actions stepwise following the instructions and information provided; (3) record and upload exercise data; (4) self-manage and monitor symptoms; (5) receive followed-up visit and communication. Correspondingly, the workstation can be used by doctors to evaluate patients’ conditions, adjust exercise prescriptions, and review exercise data to deal with abnormalities. The server provides Application Programming Interface (API) for both terminals for data interaction. Moreover, it works as a rule engine to provide decision support according to the conceptual model mentioned before.

### Experimental results

#### Step 4.1: Preliminary test

##### Preliminary effectiveness

The 6MWT, as the primary outcome related to patient’s exercise ability, showed a significant increase over time (*P* = 0.028) and 90% patients have made progress in the walking test. The patients’ average 6MWT was 473 ± 70 m before rehabilitation while it reached 506 ± 67 m after 8 weeks, which means an improvement exceed the MCID.

##### Patients’ perceptions

Overall, participants reported that this app had a positive effect on promoting exercise at home, and that they would be willing to continue using the app over an extended period.

“I personally think that exercise for rehabilitation is quite good. It plays an important role, yes, it is very good.” [Patient 3]

“Due to the cold weather of Ningxia, I used to stop outdoor morning exercises after late November. Now, I started indoor training. ……” [Patient 10]

Of course, some patients have not felt benefited.

“Generally speaking, it is very good, but I don't feel it (physical improvement). Maybe it will be better to train for a long time.” [Patient 5]

In addition, they expected to gain more health-related knowledge through this app. And the content of exercise training may need to be richer.

“… can add some psychological guidance to enhance the confidence in overcoming the disease. Knowledge of nutritional diet and health care can also be increased.” [Patient 6].

“Maybe you can add some (more difficult) training, I can easily complete these basic tasks.” [Patient 9]

#### Step 4.2: Assessment test

##### Participants characteristics

In this test (Table 5), the sample of 12 patients who evaluated this system were all male (100%) with an average age of 65 ± 6 years old (range 55–78 years). The majority had a high school education and above. 83% of participants were in Global Initiative for Chronic Obstructive Lung Disease Stage II or III (GOLD II or III) and 58% had comorbidities.

**Table 5:** Participants’ characteristics of assessment test

Patient characteristics	Value
*Demographic*	
Gender, n (%)	
Male	12 (100)
Age (years), mean (SD)	65 (6)
Education background, n (%)	
Secondary school	2 (17)
High school	6 (50)
Graduate and above	4 (33)
Current smoker, n (%)	0 (0)
Ex-smoker, n (%)	12 (100)
Comorbidities, n (%)	
Yes	7 (58)
No	5 (42)
*Clinical characteristics*	
Pulmonary function	
FEV1^a^ (L), mean (SD)	1.41 (0.51)
FEV1/Predicted (%), mean (SD)	47 (16)
FVC^b^ (L), mean (SD)	2.53 (0.56)
FEV1/FVC (%), mean (SD)	54 (12)
Global Initiative for chronic obstructive lung disease stage^c^, n (%)	
2	4 (33)
3	6 (50)
4	2 (17)
Modified medical research council, n (%)	
0	1 (8)
1	9 (75)
3	2 (17)
COPD assessment test score, mean (SD)	14 (3)
6-Min walking distance (m), mean (SD)	476 (65)

##### Clinical outcomes

The results of both the primary outcome and the secondary outcomes are shown in Table 6. Focus on the primary outcome, 6MWT showed significant difference (*P* = 0.023) over time with an improvement that exceeded the MCID, and improvement was also observed in the CCQ score (*P* = 0.006). Change in respiratory symptom (CAT score) was statistically different (*P* = 0.031) with a greater decrease of − 3 points that exceeded the MCID [40]. The mMRC grades reduced overall with grade 3 disappearing and showed a significant difference. Originally grade 1 accounted for the majority, but after rehabilitation it was mostly reduced to grade zero (Table 7). In addition, step counts, CRQ and HAD showed no statistically significant difference in this study but non-inferiority.

**Table 6:** Clinical outcomes before and after the assessment test

Outcomes	Baseline	After 12 weeks	*P* value	Change/MCID
6MWT*, mean (SD)	476 ± 65	502 ± 52	0.023	+ 26/+ 25
Steps per day, mean (SD)	7667 ± 2784	8954 ± 2336	0.187	+ 1287/
CAT*, median (Q_L_, Q_U_)	14 (12, 18)	10 (8, 15)	0.031	− 3/− 2
mMRC*, median (Q_L_, Q_U_)	1 (1, 1)	0 (0, 1)	0.011	
CCQ*, median (Q_L_, Q_U_)	13 (10,17)	11 (7,16)	0.006	− 2/− 1
CRQ, median (Q_L_, Q_U_)	21 (20, 23)	22 (20, 23)	0.105	+ 1/+ 2
HAD, median (Q_L_, Q_U_)	5 (2, 10)	4 (0, 7)	0.443	− 1/− 2

**Table 7:** Change of the mMRC grade before and after the assessment test

Patient number	mMRC before	mMRC after	Grade change	
1	1	→	0	Decreased
2	1	→	0	
3	3	→	2	
4	3	→	2	
7	1	→	0	
8	1	→	0	
10	1	→	0	
11	1	→	0	
12	1	→	0	
5	1	→	1	Unchanged
9	1	→	1	
6	0	→	1	Increased

##### Feasibility and usability

###### Compliance

**Fig. 4:**
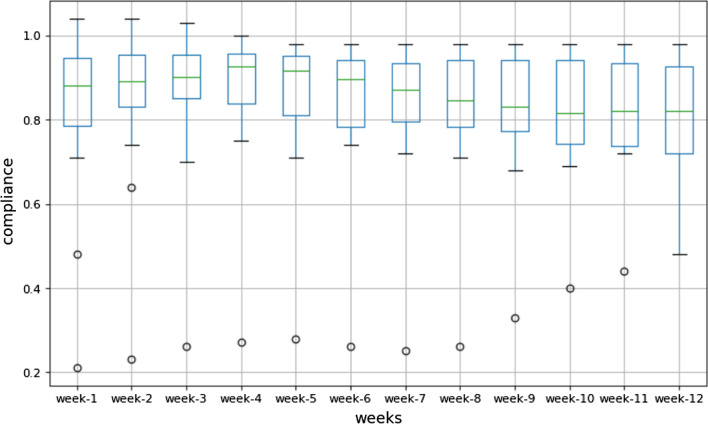
Compliance of the participants per week over the 12-weeks

The overall compliance of this study was defined as the compliance averaged across all patients and it reached 82.20% (± 1.68%). At the end of assessment test, the compliance remained at 79.33%. The boxplot (Fig. 4) depicts the distribution of the compliance of each week, with the box surrounding the 25% and 75% quantiles. The levels of compliance remained at a high level throughout the experimental period and decreased moderately and non-significantly (*P* = 0.986) at later stages.

###### Technology acceptance

**Fig. 5:**
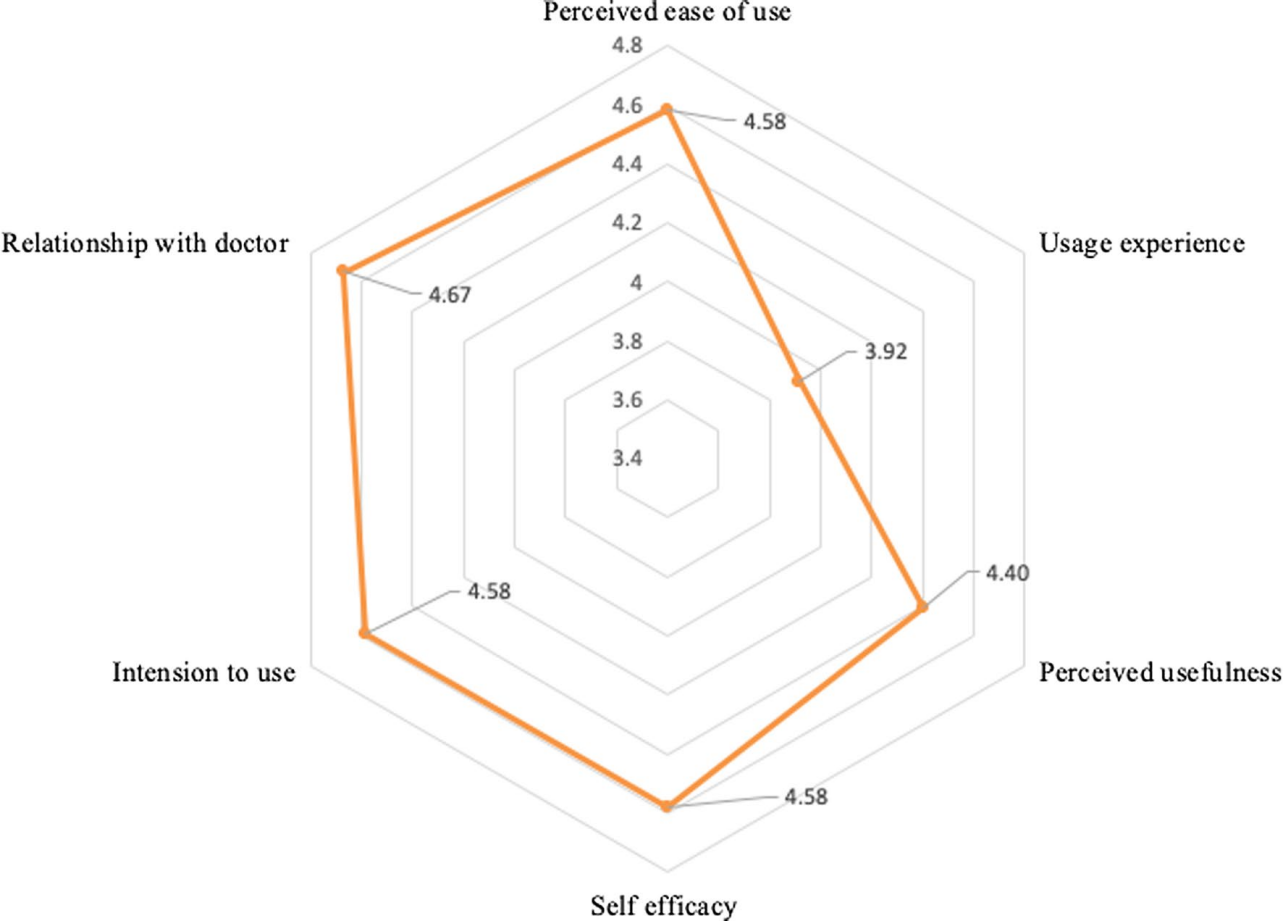
The average score of each attributes of the questionnaire for technology acceptance

Evaluation of technology acceptance by patients occurred at the end of the experiment. Users answered the questionnaire and Fig. 5 shows a radar graph of the average scores of various attributes. Scores were high across all domains, apart from the usage experience relatively (score = 3.92), where patients with lower scores indicate a poorer level of familiarity with the smartphone. Relationship with doctor was observed to affect the technology acceptance by patients in the using process (score = 4.67).

The average score for the participants was 49.3 (range 35–55, median 49.5), indicating good acceptance of the technology in this context. The interviews also revealed that the participants found the system easy both to learn and to use, illustrated by expressions such as *“It is easy to learn, I have no difficulties.” [Patient 3].*

During the interview session, only one participant reported difficulty in using the study-involved technology at the beginning of the intervention. For example, this 62-year-old man participant stated he needed some time to understand the data from the pedometer.

All of the participants would participate again. And further, all participants would recommend others to participate: *“Yes, absolutely. I will. Unfortunately, there are not so many patients with COPD around me.” [Patient 8].*

###### Functional usability

Another analysis evaluated the techniques and functions applied in this system through the average score given by the patients. When finalizing the rehabilitation program, the patient could rate these techniques with a score of 1–5 points.

The participants were satisfied with the mHealth-based intervention. Table 8 presents that the walking exercise section was the one that received the highest score average, 4.50 points. In second place was the breathing exercise section that obtained 4.33 points and the diary section was in the last place, with 4.08 points.

**Table 8:** The average score of the questionnaire for functional usability

Name of the function of the app	Average score
Breath exercise	4.33
Walk training	4.50
Diary	4.08
Message	4.25

## Discussion

### Principal findings

This study developed a home-based PR mHealth system for COPD patients, the core of which is its exercise prescription contains the standardized guidelines for PR and can adapt to patients' conditions such as exercise capacities and breathlessness and fatigue during physical work. The use of BCW in the intervention developing process offereda systematic method for designing a theory-driven intervention. In addition, our pilot study in Yinchuan demonstrated the benefits of applying mHealth technology and BCT to Home-based PR for COPD patients.

The designed exercise program provided a design scheme suitable for home environment and transmitted through mobile app. To meet the needs of patients with different improvement requirements of cardiorespiratory endurance, strength, and/or flexibility, the exercise program contained different types of exercise. To satisty different volume need, we made the three attributes that make up the volume adjustable within a certain range. Customization of patients' exercise plan can be implemented to a certain extent.

The iterative system design process made the system continuously improved based on user needs. During the iterative process, the research team made decisions to add some changes to the conceptual model of the intervention that were not thought of beforehand, such as including the exercise methods of traditional Chinese sports medicine (e.g., Tai Chi), enhancing the rule engine to modify prescription automatically. Of note, in the final version of system, we added rhythm audio to the walk training module to monitor its intensity (walking speed). Patients walked following the cueing rhythm of the audio prompt and the rhythm would be modified according to the results of the Borg scale filled out by patients after training.

Among respiratory function parameters, 6MWT scores showed significant improvement in two pilot studies. Few pulmonary rehabilitation programs based on mHealth achieved no significant improvement at 6MWT or were not mentioned at all. For example, Kwon et al. indicated that their participants had mild or moderate disease severity (GOLD 1 or 2) and their 6MWT ranged 350–400 might cause this result [17]. However, 83% of participants in this study had GOLD 2 or 3, this might explain the result. The outcome of step counts examined the potential feasibility of using an app to encourage patients with COPD to increase, or at least maintain their physical activity levels. Meanwhile, CAT, mMRC and CCQ showed meaningful improvement in our trail while CRQ did not. However, correlations between CCQ and CRQ were found and study showed that both the CCQ and CRQ are equally reliable and valid [41]. Reda et al. indicated that CRQ was a good indicator for the medium term but its responsiveness declines in the longer term and CCQ is the recommended alternative when the follow-up exceeds 26 weeks [42]. This might explain why CCQ is significant in our study but CRQ is not, as some patients participating in the assessment test had already received intervention in the preliminary test before with a longer trial period lasting for 11 months in total.

We did not find significantly improved HAD outcomes at post-intervention but non-inferiority. This may be due to the relatively healthy mental state and small sample size. The majority (7/12) of participants in our study had a score below 8 in HAD (a score above 8 indicates anxiety and depression symptoms), and only 3 participants had moderate or higher levels. Thus, the mental state of the participants is generally improved and HAD scores show a tendency of reduction but without significant change.

Feasibility and usability findings showed that participants overall were highly engaged and reported accepting the intervention. In our study, participants keep training on average for 82.2% out of the intervention period, which is comparable to findings from other studies using mHealth technology. For example, in Burkow’s work on average a 77% attendance rate was found in group exercise sessions for patients with COPD [22]. And the SMART-COPD app was used on 73% of the days on which it was deployed to a participant as Bentley reported [23]. Besides, participants had positive perceptions about technology and mHealth. Considering the result of the questionnaire, they were willing to use software for self-management and rehabilitation training out of trust to doctors and willingness to control their own disease conditions, even though they may not be tech-savvy people.

### Strengths and limitations

We showed that it is feasible to deliver a mHealth-BCT-based intervention to patients with COPD in a real-life setting over several months to promote their physical activity and capacity. Few recent studies reported promising clinical results using mHealth to conduct home-based PR in older patients. As mentioned before, Kwon et al. developed a comprehensive rehabilitation management platform as an intervention to improve physical activity and HRQL but found no meaningful improvement in 6MWT [17]. Bentley et al. reported that theory-based intervention via an app was well accepted and perceived as easy to use [23]. Their study focused on helping patients maintain physical activity after undertaking PR, which is somewhat different from the purpose of our study. In another study, Burkow et al. intervened patients using an app with functionality for a virtual peer group and visual rewards but reported limited clinical outcomes [22]. Compared with these interventions, the clinical benefits obtained by the assistance of app in this study may be attributed to several factors. First, the walking speed was exactly controlled by the tempo of audio to achieve the intended level of endurance training at home. Specific speed value was determined by the current exercise capacity of patients, which was measured by the integrated assessment of 6MWT, Borg and history compliance of previous walk tasks. In this process, the system plays an important auxiliary role in realizing different configurations of exercise prescriptions. Secondly, using smart phones, the daily record of exercise data was well monitored. The change in exercise data may draw attention to patients themselves and HCPs who observe the data on the website, and early medical intervention may be implemented to prevent acute exacerbation of COPD control. Thirdly, the long-term adherence and compliance to exercise training is the critical factor in sustaining the clinical benefit in the home setting PR program. Using internet or smartphone apps technology, a feasible and acceptable method for the monitoring of adherence can be provided, even for elderly COPD patients. In our study, timely supervision and management from HCPs are additional factors that contribute to the maintenance of compliance and clinical benefits.

Potential limitations of this study included technical issues affecting participants’ experience of using the intervention, and the impact of data completeness. Besides, present pilot studies were small, and only the first step was towards exploring feasibility of these interventions. Coupled with the impact of Corona Virus Disease 2019 (COVID-19), only preliminary and limited data on clinical outcomes are available. The statistical analyses must be interpreted with great caution given the small sample size. Further work includes conducting a randomized controlled trial with lager number of patients.

## Conclusion

In conclusion, we showed that the home-based PR mHealth system incorporating BCTs is a feasible and acceptable intervention for COPD patients, and COPD patients can benefit from the intervention. The form of intervention delivered by mHealth improved the availability of PR for COPD patients. The use of BCTs made it have great potential to be effective in increasing patients’ compliance, technical acceptance and availability, thereby enhancing feasibility. The clinical outcomes demonstrated the benefits of applying the system for COPD patients. The design process of the system offered a systematic method for designing a mHealth-based, theory-guided PR program. The system may be a useful tool for patients’ behavior change. Moreover, the proposed system played an important auxiliary role in offering exercise prescription according to the characteristics of patients. It provided means and tools for further individuation of exercise prescription in the future.

## Supplementary Information

**Additional file 1.** 4-Item usability questionnaire. This file gives the content of the questionnaire used for the measurement of functional usability.

**Additional file 1.** Examples of the questions used during the interview. This file lists 9 questions used during the interview conducted in the two pilot studies.

**Additional file 1.** BCT Taxonomy (v1): 93 hierarchically-clustered techniques. This file lists 93 hierarchically-clustered behavior change techniques, 18 of which marked with * indicating techniques identified for the development of the home-based PR mHealth system.

## Data Availability

Not applicable.

## Abbreviations

COPD: Chronic obstructive pulmonary disease
PR: Pulmonary rehabilitation
BCTs: Behavior change technologies
MRC: Medical research council
BCW: Behavior change wheel
6MWT: Six-minute walk test
CAT: COPD assessment test
mMRC: Modified British medical research council
HAD: Hospital anxiety and depression scale
MCID: Minimal clinically important difference
HRQL: Health-related quality of life
HCPs: Health care providers
COM-B: Capability, opportunity, and motivation—behavior
FEV1: Forced expiratory volume in 1 s
FVC: Forced vital capacity
CRQ: Chronic respiratory questionnaire
CCQ: Clinical COPD questionnaire
ATS/ERS: American thoracic society/European respiratory society
ACSM: American college of sports medicine
API: Application programming interface
GOLD: Global Initiative for chronic obstructive lung disease
COVID-19: Corona virus disease 2019
