# Supplementary material for: A home-based pulmonary rehabilitation mHealth system to enhance the exercise capacity of patients with COPD: development and evaluation
Source: BMC Med Inform Decis Mak. 2021 Nov 22;21:325. doi: 10.1186/s12911-021-01694-5 (PMC8607968; doi:10.1186/s12911-021-01694-5)
Supplement: Supplementary file 1 — Additional file 1. 4-Item usability questionnaire. This file gives the content of the questionnaire used for the measurement of functional usability. [file 12911_2021_1694_MOESM1_ESM.docx]

Additional file 1

4-item Usability Questionnaire

**1. The breath exercise section is useful for me.**a) Strongly agree

b) Agree

c) Neutral

d) Disagree

e) Strongly disagree

**2. The walk training section is useful for me.**

a) Strongly agree

b) Agree

c) Neutral

d) Disagree

e) Strongly disagree

**3. The diary section is useful for me.**

a) Strongly agree

b) Agree

c) Neutral

d) Disagree

e) Strongly disagree

**4. The message section is useful for me.**

a) Strongly agree

b) Agree

c) Neutral

d) Disagree

e) Strongly disagree
