# Supplementary material for: A home-based pulmonary rehabilitation mHealth system to enhance the exercise capacity of patients with COPD: development and evaluation
Source: BMC Med Inform Decis Mak. 2021 Nov 22;21:325. doi: 10.1186/s12911-021-01694-5 (PMC8607968; doi:10.1186/s12911-021-01694-5)
Supplement: Supplementary file 2 — Additional file 1. Examples of the questions used during the interview. This file lists 9 questions used during the interview conducted in the two pilot studies. [file 12911_2021_1694_MOESM2_ESM.docx]

Additional file 2

Examples of the questions used during the interview

**Objective Questions：**

1. How do you spend your day (the most common situation)? Do you often exercise? Outdoor activities (shopping, walking)?

2. Has our rehabilitation program changed your life？

**Reflective** **Questions：**

3. You have been using COPD software for some time. How do you feel about using COPD software? Can you elaborate?

4. Does this level of rehabilitation training make you feel more pressure or motivation?

5. After you use our software, can you give us a comment on which part of our software is good and which part is not good.

**Interpretive Questions：**

6. You just mentioned that doing rehabilitation exercise makes you feel pressure (motivation). Can you tell me your specific feelings in detail? For example, what kinds of exercise do you do, you will feel pressure.

7. Then tell me, where are you not satisfied with this software? We will make some adjustments and improvements. Give me some advice.

**Decision Questions：**

8. Would you like to continue to participate in our project?

9. Would you like to recommend our project to the people around you?
