# Supplementary material for: A home-based pulmonary rehabilitation mHealth system to enhance the exercise capacity of patients with COPD: development and evaluation
Source: BMC Med Inform Decis Mak. 2021 Nov 22;21:325. doi: 10.1186/s12911-021-01694-5 (PMC8607968; doi:10.1186/s12911-021-01694-5)
Supplement: Supplementary file 3 — Additional file 1. BCT Taxonomy (v1): 93 hierarchically-clustered techniques. This file lists 93 hierarchically-clustered behavior change techniques, 18 of which marked with * indicating techniques identified for the development of the home-based PR mHealth system. [file 12911_2021_1694_MOESM3_ESM.docx]

Additional file 3

BCT Taxonomy (v1): 93 hierarchically-clustered techniques

| **Grouping and BCTs** | **Grouping and BCTs** |
| --- | --- |
| **1. Goals and planning** | **9. Comparison of outcomes** |
| 1.1.  Goal setting (behavior) ***** | 9.1.  Credible source |
| 1.2.  Problem solving ***** | 9.2.  Pros and cons |
| 1.3.  Goal setting (outcome) | 9.3. Comparative imagining of future outcomes |
| 1.4.  Action planning ***** |  |
| 1.5.  Review behavior goal(s) ***** | **10. Reward and threat** |
| 1.6.  Discrepancy between current behavior and goal ***** | 10.1.  Material incentive (behavior) |
|  | 10.2.  Material reward (behavior) |
| 1.7.  Review outcome goal(s) | 10.3.  Non-specific reward |
| 1.8.  Behavioral contract | 10.4.  Social reward ***** |
| 1.9.  Commitment | 10.5.  Social incentive |
| **2. Feedback and monitoring** | 10.6.  Non-specific incentive |
| 2.1.  Monitoring of behavior by others without feedback | 10.7.  Self-incentive |
|  | 10.8.  Incentive (outcome) |
| 2.2.  Feedback on behavior ***** | 10.9.  Self-reward |
| 2.3.  Self-monitoring of behavior ***** | 10.10.  Reward (outcome) |
| 2.4.  Self-monitoring of outcome(s) of behavior | 10.11.  Future punishment |
|  | **11. Regulation** |
| 2.5.  Monitoring of outcome(s) of behavior without feedback | 11.1.  Pharmacological support |
|  | 11.2.  Reduce negative emotions |
| 2.6.  Biofeedback | 11.3.  Conserving mental resources |
| 2.7.  Feedback on outcome(s) of behavior ***** | 11.4.  Paradoxical instructions |
| **3. Social support** | **12. Antecedents** |
| 3.1.  Social support (unspecified) ***** | 12.1.  Restructuring the physical environment ***** |
| 3.2.  Social support (practical) |  |
| 3.3.  Social support (emotional) | 12.2.  Restructuring the social environment |
| **4. Shaping knowledge** | 12.3.  Avoidance/reducing exposure to cues for the behavior |
| 4.1.  Instruction on how to perform the behavior ***** |  |
|  | 12.4.  Distraction |
| 4.2.  Information about Antecedents | 12.5.  Adding objects to the environment ***** |
| 4.3.  Re-attribution | 12.6.  Body changes |
| 4.4.  Behavioral experiments | **13. Identity** |
| **5. Natural consequences** | 13.1.  Identification of self as role model |
| 5.1.  Information about health consequences ***** | 13.2.  Framing/reframing |
|  | 13.3.  Incompatible beliefs |
| 5.2.  Salience of consequences | 13.4.  Valued self-identify |
| 5.3.  Information about social and environmental consequences | 13.5.  Identity associated with changed behavior |
| 5.4.  Monitoring of emotional consequences | **14. Scheduled consequences** |
| 5.5.  Anticipated regret | 14.1.  Behavior cost |
| 5.6.  Information about emotional consequences | 14.2.  Punishment |
|  | 14.3.  Remove reward |
| **6. Comparison of behavior** | 14.4.  Reward approximation |
| 6.1.  Demonstration of the behavior ***** | 14.5.  Rewarding completion |
| 6.2.  Social comparison | 14.6.  Situation-specific reward |
| 6.3.  Information about others’ approval | 14.7.  Reward incompatible behavior |
| **7. Associations** | 14.8.  Reward alternative behavior |
| 7.1.  Prompts/cues ***** | 14.9.  Reduce reward frequency |
| 7.2.  Cue signaling reward | 14.10.  Remove punishment |
| 7.3.  Reduce prompts/cues | **15. Self-belief** |
| 7.4.  Remove access to the reward | 15.1.  Verbal persuasion about capability |
| 7.5.  Remove aversive stimulus | 15.2.  Mental rehearsal of successful performance |
| 7.6.  Satiation |  |
| 7.7.  Exposure | 15.3.  Focus on past success |
| 7.8.  Associative learning | 15.4.  Self-talk |
| **8. Repetition and substitution** | **16. Covert learning** |
| 8.1.  Behavioral practice/rehearsal ***** | 16.1.  Imaginary punishment |
| 8.2.  Behavior substitution | 16.2.  Imaginary reward |
| 8.3.  Habit formation | 16.3.  Vicarious consequences |
| 8.4.  Habit reversal |  |
| 8.5.  Overcorrection |  |
| 8.6.  Generalization of target behavior |  |
| 8.7.  Graded tasks ***** |  |

* Behavior change techniques identified to develop the home-based PR mHealth system.

This table listed all 93 Behavior Change Techniques (BCTs) sourced from Ref [1]. Specific details including definition and examples of each BCT can be found in Electronic Supplementary Materials Table 3 of Ref [1].

Reference:

1. Michie S, Richardson M, Johnston M, et al. The behavior change technique taxonomy (v1) of 93 hierarchically clustered techniques: building an international consensus for the reporting of behavior change interventions. Annals of behavioral medicine. 2013 Aug;46(1):81-95. PMID: 23512568.
